# Pharmacist provided medicines reconciliation within 24 hours of admission and on discharge: a randomised controlled pilot study

**DOI:** 10.1136/bmjopen-2016-013647

**Published:** 2017-03-16

**Authors:** Brit Cadman, David Wright, Amanda Bale, Garry Barton, James Desborough, Eman A Hammad, Richard Holland, Helen Howe, Ian Nunney, Lisa Irvine

**Affiliations:** 1 Pharmacy Department, Cambridge University Hospitals, Cambridge, UK; 2 School of Pharmacy, University of East Anglia, Norwich, UK; 3 Norwich Medical School, University of East Anglia, Norwich, UK; 4 Norwich Clinical Trials Unit, University of East Anglia, Norwich, UK; 5 School of Pharmacy, University of Jordan, Jordan

**Keywords:** medicines reconciliation, pharmacy, cost-effectiveness, randomized controlled trial, pilot study

## Abstract

**Background:**

The UK government currently recommends that all patients receive medicines reconciliation (MR) from a member of the pharmacy team within 24 hours of admission and subsequent discharge. The cost-effectiveness of this intervention is unknown. A pilot study to inform the design of a future randomised controlled trial to determine effectiveness and cost-effectiveness of a pharmacist-delivered service was undertaken.

**Method:**

Patients were recruited 7 days a week from 5 adult medical wards in 1 hospital over a 9 month period and randomised using an automated system to intervention (MR within 24 hours of admission and at discharge) or usual care which may include MR (control). Recruitment and retention rates were determined. Length of stay (LOS), quality of life (EQ-5D-3L), unintentional discrepancies (UDs) and emergency readmission (ER) within 3 months were tested as outcome measures. The feasibility of identifying and measuring intervention-associated resources was determined.

**Result:**

200 patients were randomised to either intervention or control. Groups were comparable at baseline. 95 (99%) patients in the intervention received MR within 24 hours, while 62 (60.8%) control patients received MR at some point during admission. The intervention resolved 250 of the 255 UDs identified at admission. Only 2 UDs were identified in the intervention group at discharge compared with 268 in the control. The median LOS was 94 hours in the intervention arm and 118 hours in the control, with ER rates of 17.9% and 26.7%, respectively. Assuming 5% loss to follow-up 1120 patients (560 in each arm) are required to detect a 6% reduction in 3-month ER rates.

**Conclusions:**

The results suggest that changes in outcome measures resulting from MR within 24 hours were in the appropriate direction and readmission within 3 months is the most appropriate primary outcome measure. A future study to determine cost-effectiveness of the intervention is feasible and warranted.

**Trial registration number:**

ISRCTN23949491.

**Strengths and limitations of this study**

- Pilot randomised controlled trial.
- Intervention fidelity enhanced through competency assessment of medicine reconciliation providers.
- Robust process for identifying unintentional discrepancies at each stage developed to prevent results contamination.
- Pragmatic design and therefore elements of the intervention found within the control arm.
- Limited response to request for patient data 3 months postdischarge.

## Background

Medicines reconciliation (MR) is defined by the WHO as ‘the formal process in which healthcare professionals partner with patients to ensure accurate and complete medication information transfer at the interfaces of care’.^1^ Researchers have identified unintentional error rates within hospital prescriptions on admission of between 30% and 70%^2–5^ and these can include omission of usual medicines, prescription of incorrect dosages and addition of medicines which have not been previously prescribed. The majority of these errors are believed to result from deficiencies in the MR process with such errors known to contribute to patient morbidity and mortality and increase the length of hospital stay (LOS).^6–10^

Consequently, within the UK, patient safety guidance issued by the National Patient Safety Agency (NPSA) and the National Institute for Health and Care Excellence (NICE) recommended that policies for MR should be implemented in hospitals for all adult patient admissions, and that pharmacy should be involved in the MR process within 24 hours of admission.^11^ NICE guidance on pharmacy involvement was based primarily on one randomised controlled trial (RCT) which demonstrated that the inclusion of the pharmacist in MR reduced the error rate from 44% to 19%.^2^ Data on cost-effectiveness which usually underpins recommendations made by NICE^12^ are not available and therefore whether this intervention represents an appropriate use of National Health Service (NHS) resources is unknown.

Recent systematic reviews of hospital-based MR practices consistently demonstrate a reduction in medicine discrepancies; however, this has not been shown to result in reductions in postdischarge healthcare usage.^13^ ^14^ When considering only pharmacist-led MR interventions a 19% reduction in the rate of all-cause readmissions was seen but similarly pooled data on mortality and composite readmission and emergency department (ED) visit did not find in favour of either pharmacist-led intervention or usual care.^15^

With the significant resources required for pharmacists to deliver MR services, it is not adequate to demonstrate reduction in errors (which may or may not result in adverse drug events), it is also important in resource-limited health systems to show that such investment constitutes value for money. There is currently a recognised lack of evidence supporting the cost-effectiveness of MR interventions provided by pharmacists.^16^ A model built in 2008 to estimate the likely cost-effectiveness of preventing medication error at hospital admission using MR suggested that a pharmacist-based intervention is likely to be cost-effective but was based solely on US error data and made assumptions concerning the severity of errors prevented.^17^

Despite national guidance few hospitals are providing MR as envisioned by NICE for all patient admissions.^18^ Thus, it would appear that further high-quality evidence which demonstrates cost-effectiveness is required to ensure that resources are appropriately allocated to this service in order to meet national recommendations. The most suitable approach to determine the cost-effectiveness of a complex intervention such as MR is to perform a RCT which collates data on cost from the appropriate perspective and outcomes which are most proximal to the intervention. Within the UK, it is additionally necessary to collect data on quality of life and mortality to enable the cost per quality-adjusted life year (QALY) to be estimated. Recent guidance, however, suggests that before an RCT of such nature is undertaken feasibility testing and piloting are required.^19^

## Aims

The aim of this study was therefore to pilot a RCT to inform the design of a future definitive study to determine the clinical and cost-effectiveness of pharmacy providing a full MR service on admission and at discharge. The objectives of the pilot study were to:

- Identify the most suitable outcome measure for a future definitive trial with respect to proximity and response to the intervention and quality of data obtained;
- Determine potential recruitment and retention rates;
- Develop and test the process for measuring resource usage associated with the intervention and use of other NHS.

## Methods

### Study design and location

The trial was a randomised controlled pilot study undertaken at Cambridge University Hospitals NHS Foundation Trust (CUHFT) on five adult medical wards from a range of medical specialities where patients did not routinely receive MR from a pharmacist within 24 hours of admission. One similar ward was identified as a ‘backup’, in the eventuality that one of the study wards was closed for any reason (eg, norovirus outbreak) during the recruitment period.

### Intervention

**Figure 1:**
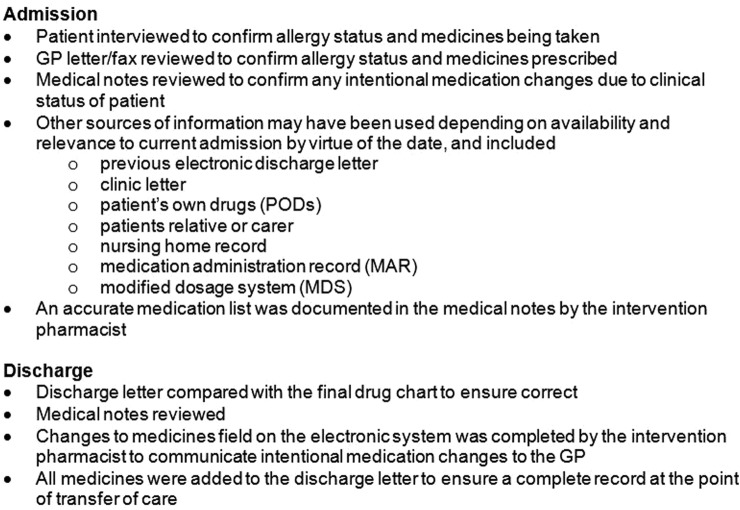
Outline of service standard operating procedure. GP, general practitioner.

A standard operating procedure (SOP) based on hospital guidelines, outlined in figure 1, was used to deliver MR by a trained MR pharmacist (MRP) within 24 hours of admission (including weekends) and at the point of transfer of care out of hospital, or as soon as possible following patient discharge from hospital to the next care provider. The five MRPs, all clinical pharmacists employed within the hospital, covered for each other's holidays, sick leave and absences wherever possible.

MRPs recorded all unintentional discrepancies (UDs), defined as differences between patient records with no identifiable rationale, they identified between the information they collated and the inpatient medication chart on admission and again any differences between the inpatient chart and discharge letter. MRPs followed up on all identified UDs to ensure that they were addressed prior to discharge.

### Control

Patients in the control arm received usual care which may or may not consist of MR and where it was provided it may not have occurred within 24 hours and could either be delivered by a pharmacist or pharmacy technician. The MRPs within the intervention arm did not deliver MR to control patients and the SOP used for study intervention purposes was not automatically followed within the control arm. For the purposes of the study all MR details regarding interventions undertaken within the control arm were recorded and costed.

### Intervention fidelity

To enhance intervention fidelity all MRPs were observed by the principal investigator on at least three occasions to confirm adherence to the SOP. All MRPs had provided MR to more than 30 patients in the year previous to delivering the intervention for the trial.

### Recruitment

A recruitment target of 200 patients was set for the 9-month pilot phase. Study wards were visited every morning by the research assistant (RA) during the study period to identify potential participants. The nurse in charge of the ward confirmed that it was appropriate for the patient to be approached to be consented to participate in the study. Patients were recruited based on the following inclusion and exclusion criteria.

#### Inclusion criteria

- Adult (≥18 years of age);
- Admitted with at least one prescribed medicine to one of the five medical wards;
- Patient had not already received MR from the pharmacy team as part of routine pharmaceutical input at the time of recruitment;
- Identified from hospital computer system as having been admitted straight from the ED to one of the five participating wards within the previous 24 hours.

With the intervention required to be delivered within 24 hours of admission, patients were given a maximum of 2 hours to consider whether they wished to participate. Intervention patients received MR in accordance with the SOP. Information obtained by the RA for the purposes of the study was given to the MRP prior to the visit to prevent duplication of effort and to ensure that the patient was not interviewed for the same information twice.

Randomisation was performed using the Norwich Clinical Trials Unit automated service with patients stratified by ward. When wards were later closed for infection control reasons, participants on the ‘backup’ ward were randomised and stratified as if they had entered the closed ward.

Patients randomised to the control group received usual care; this may have included elements of MR by members of the pharmacy team and in some cases may have occurred within 24 hours of admission but postrandomisation. While this may have affected patients in the control arm an intention-to-treat analysis was performed and consequently for the purpose of the analysis patients remained in their allocated arm.

### Outcome measures

Although undertaken as a pilot study with study aims to identify the most suitable outcome measure, LOS was nominally selected as the primary outcome measure for this pilot trial. Secondary outcome measures were unplanned (emergency) readmission at 3 months, quality of life (EQ-5D-3L) and UDs.

### Data collection

To enable comparison of intervention and control groups, age, gender, primary reason for admission, all comorbidities and the admission ward was recorded. Additionally consented patients or their third party consultees completed a quality of life score (EQ-5D-3L)^20^ on admission, including the related visual analogue scale (VAS).

LOS, reported in hours, was calculated as the difference in time from arrival at the hospital to the time of discharge as recorded in the hospital information support system (HISS). Unplanned readmissions to the intervention hospital within the 3 months postdischarge were also obtained from HISS. EQ-5D-3L responses were also obtained via postal survey 3 months postdischarge, allowing the calculation of QALY^21^ in the subsequent economic evaluation.

### UD identification

To enable the identification of UDs the following information was photocopied by the research assistant (RA) for all consented patients (both intervention and control) and stored securely:

- All versions of the inpatient medication chart(s) and discharge letters;
- Medical notes during admission;
- General practitioner (GP) medication list on admission;
- GP medication list at 3-month postdischarge (when received from GP surgery);
- Any additional medicines-related information brought in by the patient on admission, for example, copies of labels from patient medicines, handwritten or typed medicine lists.

The RA was a trained nurse and consequently was experienced in medical data collation and extraction.

Three months postdischarge the stored information was used to develop an ‘accurate medication list’ for the control arm patients by the research team on admission and at discharge. These were then compared with the inpatient chart on admission and discharge letter to identify any discrepancies. Medical notes were subsequently reviewed, unblinded to group allocation, to enable differentiation between those which were UDs which could not be explained from the information available and those which were intentional.

The GP medication list obtained 3 months postdischarge (where available) was used to enable the identification of discrepancies which still remained at 3 months. Without access to GP medical notes it was not possible to establish whether identified discrepancies were unintentional.

All potential discrepancies which were identified at the 3-month point as having translated into the patient notes were reported to the patient's GP.

### Resource use

The time taken by the pharmacy team to deliver the MR was monitored in the intervention and control group (if applicable). Additionally, the following items were requested in both groups:

- Time in hospital (LOS);
- Medication (in patient medication and GP medication list at 3 months);
- Rehospitalisations;
- Other healthcare contacts.

With the exception of the final item, which included all health professional contacts and was requested from the participant at 3 months postdischarge, these were extracted from medical records.

### Sample size calculation

As a pilot study a formal power calculation was not performed. The consequences for the precision of the primary outcome variable (LOS) of the choice of sample size were however estimated. Summary statistics on LOS taken from a study undertaken at St James Hospital, Dublin^22^ gave quartiles for two groups as (3, 7, 5) and (2, 5, 12) days. To derive an estimate of variability from this, an underlying log-normal distribution was assumed, which is consistent with the position of the medians, and its SD estimated using the geometric mean of the ratios between the upper and lower quartiles. This produced an estimated SD of 1.26 which implied that a comparison between two groups of 100 patients would provide an expected half-width for the 95% CI of the difference between group means (on the natural log scale) of 0.35. Translated back to the CI for the ratio of average LOS between the intervention and control groups would extend by a factor of about 1.4 on either side of the point estimate.

### Data analysis

As a pilot study descriptive statistics were used to determine the suitability of the different outcome measures and report the variation in the differences in order to determine a sample size for a future RCT. Similarly, completion rates are reported for each source of resource use data and the EQ-5D-3L.

The primary outcome variable, LOS, was reported using median, arithmetic mean and geometric mean. The rate of Trust readmissions, Trust emergency readmissions and mortality are also reported for both arms, along with the mean change in the VAS from the EQ-5D-3L.

## Results

**Figure 2:**
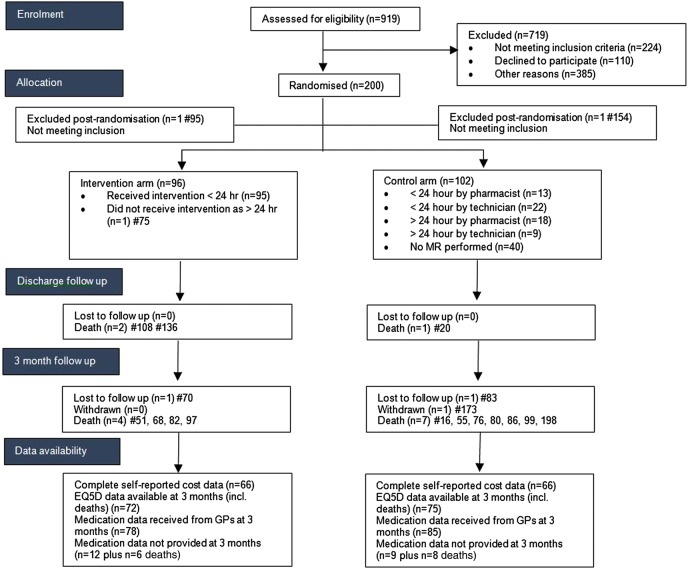
Consort diagram. GP, general practitioner; MR, medicines reconciliation.

Nine hundred and nineteen patients were assessed for eligibility of which 224 did not meet inclusion criteria. Two hundred out of 310 patients who were subsequently approached by the RA consented to take part in the study (figure 2). Of those patients identified as potentially eligible but not approached this was primarily because either the nurse in charge of the ward advised that the patient was not suitable to be approached or that the end of the 24-hour window for intervention was due to expire.

Recruitment took place between July 2012 and April 2013 (9 months and 2 weeks), resulting in a recruitment rate of 5.2 patients per 7 days.

There was one postrandomisation exclusion in each arm (they were subsequently found not to meet the inclusion criteria), and were accordingly excluded from all analyses. Table 1 summarises the characteristics of the remaining 198 participants (96 intervention 102 control). The groups were broadly comparable.

**Table 1:** Comparison of demographics at baseline

Demographic	Measure	Intervention (n=96)	Control (n=102)
Female	N (%)	45 (46.9)	60 (58.8)
Age (years)	Mean (SD)	67.6 (19.0)	65.4 (20.2)
Regular medicines	Mean (SD)	5.84 (4.07)	6.67 (4.64)
As required medicines	Mean (SD)	0.85 (2.08)	0.95 (2.53)
Ward			
1	N (%)	26 (26.8)	27 (26.2)
2	N (%)	30 (30.9)	30 (29.1)
3	N (%)	10 (10.3)	9 (8.7)
4	N (%)	14 (14.4)	16 (15.5)
5	N (%)	16 (16.4)	14 (13.5)
6	N (%)	1 (1.03)	7 (6.8)
EQ-5D quality of life (visual analogue scale)	Mean (SD)	55.9 (23.2)	54.7 (23.5)
Reason for admission			
Lower respiratory tract infection	N (%)	9 (9.2)	11 (10.7)
Troponin negative chest pain	N (%)	7 (7.2)	4 (3.9)
Heart failure	N (%)	2 (2.1)	5 (4.9)
Exacerbation chronic obstructive pulmonary disease	N (%)	4 (4.1)	3 (2.9)
Other	N (%)	73 (75)	79 (76.7)

One patient in the intervention arm did not receive the MR as he was not on the ward when the pharmacist visited. Data were erroneously not collected for this patient postrandomisation. The time taken by the pharmacist to deliver the intervention was recorded for the remaining 95 intervention participants, where the mean total time was 48.6 min (range 2–195 min). In the control group 62 (60.8%) participants received some form of MR, 31 (30.4%) from a pharmacist (mean reported time 15 min) and 31 (30.4%) from a pharmacy technician (mean reported time 12 min). Twenty-five (24.5%) control patients received MR within the 24-hour window.

During the initial hospital admission 3 participants died, with a further 11 deaths during the 3 months follow-up period (see figure 2). In total six intervention and eight control patients died during the trial period (p=0.78, Fisher's exact). Additionally, in the control arm, one participant was lost to follow-up (address not known) and one participant withdrew. After taking account of the 1 patient who did not receive the MR and a further loss to follow-up in the intervention arm, this left a total of 88 available cases in the intervention arm and 92 available cases in the control arm at final assessment point. In terms of outcomes, there were complete data available on LOS and readmission data.

Table 2 provides a summary of the UDs which were identified at each stage. Sixteen (16%) intervention patients had no discrepancies at admission and discharge, compared with 12 (12%) control patients. Overall, two UDs were known to remain at discharge in the intervention arm compared with 268 in the control group. Neither of the UDs in the intervention arm identified at discharge was present at 3 months.

**Table 2:** Comparison of UDs

	Intervention			Control		
Unintentional discrepancies	No. UDs	No. patients	No. per patient	No. UDs	No. patients	No. per patient
Admission	255	95	2.80	309	102	3.0
Resolved during hospital stay	250	95	2.74	Unknown		
Remaining at discharge	2	91	0.02	268	99	2.71

UDs, unintentional discrepancies.

One hundred and fifty-four UDs identified in the control arm were potentially related to medicines to be prescribed postdischarge, that is, the remainder related to medicines prescribed during admission only. Owing to the limited number of GP records provided at 3 months data were only available for 82 (53.2%) UDs in the control arm at 3 months and 37 out of the original 154 (24%) were found from the medical records provided to have been propagated into the patient notes.

Table 3 provides a comparison of patient outcomes for the intervention and control group. The results suggest that LOS, mortality and rehospitalisation rates tend to be lower in the intervention arm although statistical significance was not demonstrated as 95% CIs overlapped. Based on those who responded, both groups had a higher mean quality of life (based on the VAS) at the 3-month follow-up with improvement higher in the control arm. This difference between groups was not significant.

**Table 3:** Comparison of outcome measures

Outcome	Measure	N	Intervention	N	Control	Mean difference (SE of the difference)
Length of stay (hours)	Geometric mean (95% CI)	95	99.6 (76.59 to 129.63)	102	109.3 (87.0 to 137.3)	
	Median (range)	95	94.0 (12–1077)	102	117 (13–1546)	
	Arithmetic mean (SD)	95	224.8 (293.1)	102	203.9 (246.8)	20.84 (38.75)
Hospital readmissions	N (%)	95	30 (31.6)	101	37 (36.6)	
Hospital readmissions (emergency)	N (%)	95	17 (17.9)	101	27 (26.7)	
Mortality	N (%)	95	6 (6.3)	95	8 (7.8)	
Quality of life visual analogue scale change from baseline (high score better)	Mean (SD)	63	5.64 (23.6)	68	7.15 (26.2)	1.51 (4.36)

With regard to the response rates for the resource use data, there were complete data available (for those on whom it was requested) for LOS, medication data as part of the original admission, readmissions (to the same hospital) and the intervention pharmacist times. Of the 62 controls for whom pharmacist/pharmacy technician review occurred, the times were missing for N=27. At 3-month follow-up, medication data were retrieved from GPs for 86 participants (94.5% of those from whom it was requested) in each arm and 133 participants completed and returned both the EQ-5D-3L and the health resource use questionnaires (66 intervention, 67 control; 73.5% of those requested from all living participants).

Based on the pilot data, we calculated that for a full trial, 1120 patients would need to be recruited to detect a 6% reduction (conservative assumption, one SE below 9% reduction seen) in 3-month unplanned readmission rates from a starting point of 26% with 90% power, using a 5% significance level and assuming 5% loss to follow-up.

## Discussion

The results from this study which was performed to inform the design of a future RCT suggest that even though MR activities are taking place, such a trial is feasible with reasonable recruitment and retention rates and that both cost and outcome data can be effectively obtained.

We consider that emergency rehospitalisation within 3 months would be the most appropriate primary outcome measure for such a trial, as unlike the other outcome measures tested it reflects all of the MR activity which occurs in secondary and primary care. Furthermore, data collection was complete and this is a patient-orientated outcome, unlike medication errors which while representing a patient safety issue are a measure of the prescribing process.

Owing to the need to recruit patients before they received MR as usual care, this reduced the generalisability of the sample with all of those who had already received MR being automatically excluded. This was further compounded by recruitment activities which were focussed towards mornings.

With a hospital requirement that all patients receive MR within 24 hours of admission, it is unsurprising that two-thirds of the control arm received some form of MR during their hospitalisation, a quarter of whom received this within 24 hours. There is no specific requirement for MR on discharge, however, and this may explain some of the differences seen between the two groups. The majority of discrepancies identified in the control arm by the researchers were found not to have been resolved and therefore reasons for the relative ineffectiveness of the control arm MR requires elucidation through a detailed process evaluation.

The average time spent on the intervention found within this study was very similar to that reported in a MR time and motion study^23^ but three times greater than that in the control arm. The study SOP required pharmacists to undertake initial MR, follow-up on all interventions to ensure that discrepancies had been addressed, assess all discharge letters for accuracy and correct them. Patients in the control arm frequently did not always receive all four elements and this probably explains most of the difference in time provided. It has been suggested that organisations are probably unlikely to repeat the benefits from MR services reported in the literature if there are deficiencies in intervention intensity and breadth.^24^ The results from the control arm of this study support this assertion and suggest that if MR of a similar nature to that seen in the intervention arm was to be shown to be cost-effective, then this would require significantly more pharmacist time.

The proportion of patients screened for eligibility and eventually recruited to the study was 20% and this could have been improved by increasing the number of weekends covered (from 80% to 100%). Although some patients were not suitable for inclusion in the study as they had already been reviewed by a member of the pharmacy team prior to being approached by the RA, there was a sufficient number of patients available for recruitment.

When identifying UDs, we have assumed that the MRP generated list in the intervention arm and the RA generated list in the control arm were accurate. Both are unrealistic assumptions and ideally within a definitive study all data should have been reviewed independent of the service and blinded to group allocation. The unblinded identification of MRs and inability to confirm intentional or unintentional nature of errors in many instances also means that the data on UDs must be treated with further caution.

The process of collating all medicines-related data at different time points and sealing it until 3 months postdischarge provides an opportunity to identify discrepancies without adversely affecting the intervention. While the identification of UDs should be undertaken blind of allocation, the resources required for this may not be warranted when considering that the intentional or unintentional nature of the discrepancy cannot always be accurately determined.

Considering evidence published poststudy completion, limiting the study population to those over the age of 70 and including a postdischarge telephone call as part of the intervention may have further enhanced the intervention.^13^

In line with previous research, the intervention prevented a large number of unintentional medicines-related discrepancies both during admission and postdischarge.^3–6^ The data obtained suggest that just less than a quarter of UDs identified at discharge were found to actually translate into primary care records at 3 months. While reasons for non-translation were not elucidated, the research suggests that the use of number of UDs at discharge as an outcome measure may overemphasise the problem.

Not all primary care medication lists were made available to researchers and approaches to addressing this will require consideration for a future definitive study. Using data on ‘unplanned readmission at 3 months' would seem to be the most appropriate primary outcome measure for a future definitive study as it is a patient-orientated outcome which is most likely to reflect the effect of errors which occur at all stages of the process. Similar to hospital readmission, LOS is a cost which would be captured in any cost-effectiveness analysis. LOS was however found to be largely skewed by a small number of individuals who were admitted for extended periods. Furthermore, it is not affected by errors which translate into primary care, that is, does not reflect the full impact of the service. Within the UK, hospitals are penalised for unplanned readmissions within 1 month of discharge and therefore collection of this data may also be warranted for a UK-based definitive study.

Unplanned readmission has been used as a primary outcome measure within other similar MR trials.^25–29^ While differences in unplanned readmission at 3 months have been demonstrated,^28^ ^29^ this has frequently not been the case at either 1 or 6 months.^25–27^ Within the first month, patients may still be using the medication they were discharged with and consequently this may lessen the impact of errors on the discharge letter resulting from incorrect translation into primary care. Kwan *et al*^14^ after systematically reviewing the literature suggest that unplanned readmission data should probably be collected for more than 30 days postdischarge. Unplanned readmission at 6 months will be affected by more factors unrelated to the index admission than at 3 months and therefore it may be more difficult to identify the impact of MR intervention using this outcome measure.

Quality of data collection with respect to LOS, mortality and readmission rates was high as this information was available from hospital records. Resource usage data and quality of life scores were, however, only available for two-thirds of participants at follow-up. Consequently researchers powering a definitive study on readmission rate will need to consider the possibility of there being more missing data for the cost-effectiveness analysis and imputation of missing data^30^ may be necessary to address this.

 This study was a pilot study and was not designed to obtain a definitive answer to whether MR provided to all patients within 24 hours of admission was cost-effective. However, it was conducted as an RCT, conforming to expected standards and consequently it can be assumed that it would be possible to perform a full-scale RCT in the future. An internal pilot would be warranted in such a trial as the trial should be multicentre in nature and consequently local variations in recruitment rates and service delivery would require identification and appropriate local adaptation. Additionally, costs related to service delivery may differ between settings and consequently time for service delivery would require estimation.

The pilot study has shown that it is feasible to perform an RCT to determine the effectiveness and cost-effectiveness of a pharmacist MR within 24 hours of admission and again at discharge. This study has demonstrated that this form of intervention does appear to reduce medication errors at discharge, and may reduce LOS and hospital readmissions. We consider that unplanned readmission at 3 months is the most suitable primary outcome measure but LOS, errors, mortality and quality of life should be captured.

Additionally, a thorough process evaluation is warranted in order to provide a more complete understanding of the intervention. Pharmacists delivering the intervention arm within a definitive study should follow an SOP and be trained to undertake the role to ensure standardisation in delivery. While the results of this pilot emulate other studies where prescribing errors were reduced in the intervention arm, there is an additional cost associated with providing the intervention and therefore high-quality evidence from a multicentre RCT is now needed to determine both its effectiveness and cost-effectiveness.
